# An AI-Enabled Bias-Free Respiratory Disease Diagnosis Model Using Cough Audio

**DOI:** 10.3390/bioengineering11010055

**Published:** 2024-01-05

**Authors:** Tabish Saeed, Aneeqa Ijaz, Ismail Sadiq, Haneya Naeem Qureshi, Ali Rizwan, Ali Imran

**Affiliations:** 1 AI4Networks Research Center, Department of Electrical & Computer Engineering, University of Oklahoma, Tulsa, OK 74135, USA; haneya@ou.edu (H.N.Q.); ali.imran@ou.edu (A.I.); 2 James Watt School of Engineering, University of Glasgow, Glasgow G12 8QQ, UK; ismail.sadiq@glasgow.ac.uk; 3 AI4lyf, Bahria Town Lahore, Lahore 54000, Pakistan; dr.rizwan@ai4lyf.com

**Keywords:** cough, COVID-19, confounding variables, spectrograms, diagnosis, deep-learning, c-GAN, respiratory diseases

## Abstract

Cough-based diagnosis for respiratory diseases (RDs) using artificial intelligence (AI) has attracted considerable attention, yet many existing studies overlook confounding variables in their predictive models. These variables can distort the relationship between cough recordings (input data) and RD status (output variable), leading to biased associations and unrealistic model performance. To address this gap, we propose the Bias-Free Network (RBF-Net), an end-to-end solution that effectively mitigates the impact of confounders in the training data distribution. RBF-Net ensures accurate and unbiased RD diagnosis features, emphasizing its relevance by incorporating a COVID-19 dataset in this study. This approach aims to enhance the reliability of AI-based RD diagnosis models by navigating the challenges posed by confounding variables. A hybrid of a Convolutional Neural Networks (CNN) and Long Short-Term Memory (LSTM) networks is proposed for the feature encoder module of RBF-Net. An additional bias predictor is incorporated in the classification scheme to formulate a conditional Generative Adversarial Network (c-GAN) that helps in decorrelating the impact of confounding variables from RD prediction. The merit of RBF-Net is demonstrated by comparing classification performance with a State-of-The-Art (SoTA) Deep Learning (DL) model (CNN-LSTM) after training on different unbalanced COVID-19 data sets, created by using a large-scale proprietary cough data set. RBF-Net proved its robustness against extremely biased training scenarios by achieving test set accuracies of 84.1%, 84.6%, and 80.5% for the following confounding variables—gender, age, and smoking status, respectively. RBF-Net outperforms the CNN-LSTM model test set accuracies by 5.5%, 7.7%, and 8.2%, respectively.

## 1. Introduction

Respiratory diseases (RDs) are among the most common chronic diseases and a primary cause of morbidity and mortality, imposing an immense burden on health worldwide [1]. COPD, acute lower tract infections, asthma, lung cancer, tuberculosis, and the recent COVID-19 infection are considered the most common acute respiratory diseases [2]. Millions die due to chronic RDs every year because of the lack of timely and accurate diagnosis [3]. The reliable diagnostic tests that are available for the detection of RDs are mostly laboratory-based, expensive, and time-consuming. For instance, a reverse transcriptase polymerase chain reaction (RT–PCR) test specific to SARS-CoV-2 virus is routinely used for reliable detection. This test cannot be used for continuous monitoring, since it can take up to 2 days for determining the diagnosis [4]. Additionally, to rule out the possibility of false negative results, repetitive testing may be required. This underscores the pivotal need for devising alternative methods for rapid, cost-effective, and accurate diagnosis of RDs. Moreover, there exists a dearth of reliable instantaneous screening tools that can detect the RDs at their onset [5]. Such tools are necessary for curbing the spread of the contagious RDs and averting the deterioration of public health.

As a pragmatic solution to this momentous problem, cough acoustic signals can play a crucial role in monitoring and detecting RD more effectively. Recent surveys [5,6] indicate that individuals with respiratory illness exhibit distinct features in their acoustic signals, representative of the vocal tracts, which can be extracted. Hence, features extracted from cough can help to continuously monitor and establish the health status of individuals at risk of RDs such as tuberculosis, asthma, COPD, and COVID-19 [2,5,7]. Therefore, a smart, accessible, and cost-effective self-monitoring framework for continuous cough monitoring and quick disease diagnosis [7] can be devised by incorporating the recording of cough sounds and implementing AI-based cough processing solutions [8,9].

Our seminal work on AI-enabled cough-based disease diagnosis has further triggered this interest, where RDs such as COVID-19 can be accurately diagnosed using cough. We demonstrated that it is possible to design advanced machine learning (ML) models to (1) discern cough from non-cough sounds recorded via a smartphone app, and (2) detect RDs such as COVID-19 infection, bronchitis, bronchiolitis, and pertussis from cough sounds recorded via the same app [10,11]. In recent years, a large number of independent studies have proposed solutions for digital cough monitoring and collection for timely diagnosis of RDs [12,13,14,15,16,17]. The current literature on SoTA cough-based RD detection leverages several ML and deep learning (DL) algorithms that can classify various temporal, spectral, and statistical cough features, including those that are perceptually indistinguishable to the human ear [5]. The cough-acoustic AI-based RD diagnosis models include traditional methods like support vector machines [18] for the classification of croup from pneumonia, asthma, bronchiolitis; random forest model used for COVID-19 diagnosis [19]; logistic regression model for the classification of croup and pneumonia [12]; and gradient boosting for COVID-19 classification [20,21]. Several DL models have also been leveraged for cough-based RD diagnosis including CNNs [8] for COVID-19 diagnosis and for croup, pertussis, bronchitis, and asthma classification [22]. Deep neural networks and spiking neural networks have also been used for the classification of COVID-19, asthma, bronchitis, and pertussis with remarkable accuracy [16,23]. Hybrid, e.g., CNN-LSTM, and ensemble models are also implemented in recent studies for RD diagnosis, achieving high accuracy [17,24,25,26,27,28,29,30,31].

In the rapidly advancing field of AI-enabled automated cough sound monitoring and digital disease diagnosis, remarkable performance metrics have been achieved, as evidenced by several notable studies [8,10,14,32,33,34,35,36]. However, a critical concern remains unaddressed in the majority of these studies: the potential impact of confounding variables and data biases on the performance of the AI models they employ. These models tend to overestimate their classification performance and overfit to the training data biases, while falling short in terms of proper validation and generalization to unseen data. A recent investigation by the University of Cambridge in the UK underscored this glaring deficiency within a substantial body of research dedicated to accurately detecting and diagnosing COVID-19, highlighting the oversight of confounders in the evaluation of AI frameworks [35]. The root issue is that confounding variables can distort the apparent relationship between input features and diagnostic outcomes, leading to erroneous predictions [37,38]. For instance, studies aiming to distinguish individuals with a disease from healthy controls often face the challenge of dealing with a substantial age difference between the two groups. In such cases, the AI model may inadvertently learn associations primarily influenced by age disparities rather than the genuine disease-related biomarkers and features, thus severely hindering its ability to generalize its findings. Importantly, these confounding factors can include a range of biases, such as those related to age, gender, race, and medical history, all of which can introduce systemic inaccuracies and pose potential threats to equitable healthcare assessment outcomes. Addressing these challenges is crucial for improving the reliability and fairness of AI models in the context of disease diagnosis and monitoring. The findings of our study highlight the importance for future studies to consider accounting for the effects of the confounding variables, similarly to RBF-Net, so that the reported results will be a realistic representation of classification expected in a real-world scenario.

To address this limitation in the existing studies on cough-based diagnosis, we propose an end-to-end generalized RD Bias-Free Network (RBF-Net) and evaluate its efficacy on a COVID-19 dataset. To the best of the authors’ knowledge, this is the first study that proposes a framework that is robust to the confounding variables for COVID-19 diagnosis, thus providing realistic and generalized performance. The proposed RBF-Net framework contains a bias predictor module that helps in identifying features from the cough recordings that are statistically invariant to confounding effects and mainly characterized by the effects of COVID-19, using an adversarial learning technique [39].

The contributions of this work are summarized as follows:

- In contrast to the majority of previous studies that rely on crowd-sourced cough audio databases for training AI models, this study curated a cough data set containing COVID-19 infection status. For each participant, the curated data set includes cough recordings tagged with reliable RT-PCR information and collected in a clinical setting. Hence, the data set used has extremely reliable ground truth labels, resulting in the accurate training of RBF-Net.
- To demonstrate the impact of confounding variables, we train a SoTA DL model on different splits of biased training scenarios from the cough data set based on gender, age, and smoking status. Moreover, we present an insightful analysis on how model performances are often overestimated due to the underlying biased distribution of the training data and the use of cross-validation technique.
- To overcome the impact of biases, we present an RBF-Net that learns features from cough recordings that are impacted by COVID-19. We perform a comparative analysis of the existing SoTA CNN-LSTM model with RBF-Net and demonstrate the improvement achieved by the proposed RBF-Net in terms of different performance metrics.

The remaining contents of this paper are organized as follows: Section 2 discusses the details of the cough data acquisition and its pre-processing. Section 3 presents the proposed RBF-Net architecture, and Section 4 describes the methodology adopted for the study. The results for classification on data with various biases using the proposed RBF-Net and the existing SoTA CNN-LSTM are explained in Section 5. Section 6 discusses the impact of our work and future clinical deployment, and acknowledges some limitations of this work. Finally, the conclusions of the study are given in Section 7.

## 2. Cough Data Acquisition and Pre-Processing

We have collected a corpus of high-fidelity audio data containing cough acoustics of normal and COVID-19 diagnosed patients. The notable feature about the data set is that rather than being collected through crowd-sourcing, it was curated to have a valid tagged RT-PCR test result for each sample. The audio data sample acquisition was performed during the time period of April–October 2020, in collaboration with Dow Medical College, Pakistan. For this research, the cough data samples were recorded from the subjects through our in-house developed AI4COVID app [40], under the supervision of trained nurses, using one smartphone model to avoid the impact of device variability. Each participant recorded multiple coughs in a recording sample, with each recording duration varying from 3 to 12 s. An informed consent was obtained from each participant prior to acquiring the cough data. The guidelines to interact with the potential COVID-19 patients recommended by the WHO were strictly followed at all stages of the cough data collection. For instance, the healthcare professionals wore personal protective equipment (PPE) and followed a protocol for the smartphone disinfection before and after the sample was recorded. In total, the data were collected from 1094 participants with positive RT-PCR test results, labelled as COVID-19 positive, and 3761 participants with negative RT-PCR test results, labelled as normal. In addition, the anonymity of the users was preserved at all stages during the data collection.

**Figure 1:**
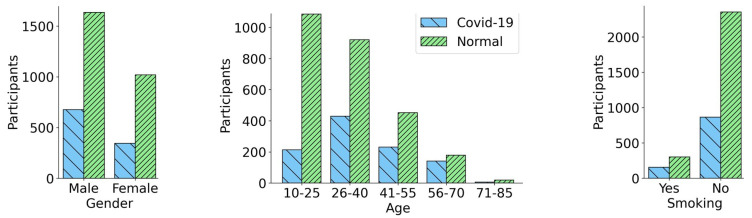
Bar plots representing the detailed demographic and smoking status statistics for the selected participants after the process of data cleaning. A total of 1022 COVID-19 positive participants and 2656 normal participants were finalized.

**Figure 2:**
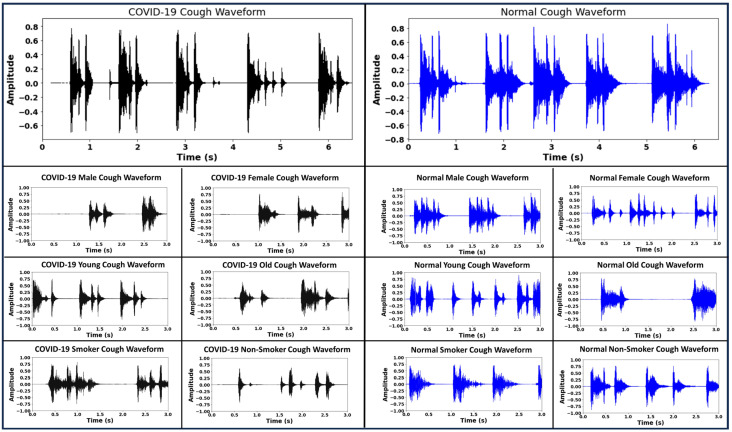
Sample waveforms for each target class, i.e., COVID-19 and normal. The waveforms contain information regarding the gender, age and smoking status that can cause a classifier to learn inaccurate representation for a cough recording with respect to a target disease label.

Once the data acquisition process was complete, we performed the cough sound pre-processing steps, including noise removal, using the Audacity software package [41]. The mono-channel cough data were sampled at 44.10 kHz before being stored as pulse-code modulation (PCM) WAV files. Silent periods at the beginning and at the end of each cough recording were clipped out. After the initial pre-processing, only the cough recordings that were longer than 2 s in duration were considered for further analysis. Thus, at the completion of the pre-processing step, we had a total of 1022 COVID-19 positive samples and 2656 normal samples. Figure 1 provides a summary of the demographics for the individuals constituting the cough audio data set. It illustrates the number of COVID-19 and normal participants with respect to their demographics, including gender, age, and smoking status. It can be observed from Figure 1 that both normal samples and COVID-19 positive samples were skewed towards the male gender, i.e., the male cough samples were almost twice as numerous compared to the female samples. Similarly the age of the subjects in the data set fell in a broad age range from 10 to 85 years, with a significant number of the cough samples belonging to the younger and middle-aged population, 18–50 years old. A small fraction of the participants were smokers, 865 subjects who tested COVID-19 positive, and 2355 normal participants were non-smokers. On the other hand, there were a total of 156 COVID-19 positive smoking participants and 301 normal smoking participants. Figure 2 demonstrates the sample waveforms from the COVID-19 and normal classes. Respiratory disease affects the lungs and results in changes in the acoustic signature of the cough sound. These changes are not always clearly identifiable on inspection. Deep neural networks trained on audio data comprising coughs from respiratory disease can learn to identify cough acoustic features characterizing the respiratory disease [5]. In addition to disease status, the cough waveforms have information like gender, age and smoking habits encoded within them that can lead the classifier to learn inaccurate representations for the disease labels. By incorporating the bias-free mechanism, the RBF-Net learns to disassociate the effects of the confounding factors like gender, age and smoking status from the disease status when classifying the cough recording. Further details are given in Section 4.

## 3. RBF-Net Architecture

**Figure 3:**
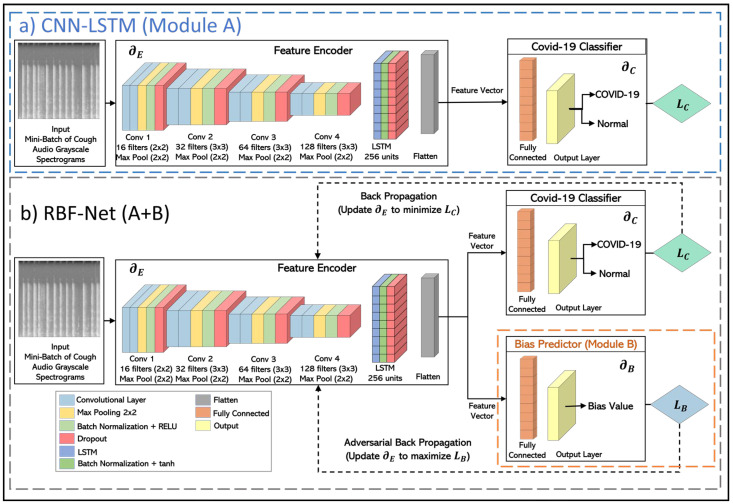
Overview of the architecture of the RBF-Net framework. (**a**) CNN-LSTM model architecture is shown, consisting of feature encoder (convolutional and LSTM blocks) and a COVID-19 classifier (fully connected layers with ReLU activation). (**b**) In the RBF-Net, we have an additional bias predictor module (fully connected layers with ReLU activation) that predicts bias from the feature vector. The losses, *L_C_* and *L_B_*, are back-propagated to train the encoder through a min-max game similarly to c-GAN, so that the extracted features are invariant to the confounding variables.

In this section, we present the detailed architecture of the proposed RBF-Net with a focus on COVID-19 classification. The CNN-LSTM model forms the main skeleton of the proposed framework architecture. The CNN-LSTM has proved to be the SoTA spectrogram-based COVID-19 classification DL model and has been widely used for classification tasks by the research community [17,24,25,26,27,28,29,30,31]. We used this SoTA DL technique as both the benchmark for our framework and also the building block for the feature encoder module of the proposed RBF-Net. The architectural details of the implemented CNN-LSTM model are shown in Figure 3a.

The CNN-LSTM architecture can primarily be divided into two blocks. The first block (feature encoder) uses a CNN architecture that receives grayscale spectrograms constructed from cough recordings as an input of shape 224 × 224. Then, the most relevant and informative features are extracted by the convolutional layers. These features are converted to the feature maps, which are passed on to the LSTM block, where the deep features that have high temporal correlation are selected to capture the more useful patterns. In the second block (COVID-19 classifier), a simple fully connected layer is used for the feature learning and COVID-19 classification. Both of these blocks are trained through the COVID-19 classification loss (*L_c_*), which is chosen to be the binary cross-entropy loss. The *L_c_* is backpropagated in a manner that the model parameters of the feature encoder (*∂_E_*) and COVID-19 classifier (*∂_C_*) are tuned to minimize.

Although this model works extremely well in learning the differences between the two classes in the target variable (COVID-19 or normal), as shown in Section 5, it can not nullify the impact of the confounding variables. This model is prone to be affected by the impact of biases in its learned features; thus, the model cannot be truly representative of COVID-19’s impact on the cough sounds. To address the underlying biases and confounding variables in the data distribution, we made modifications to the CNN- LSTM inspired by the recent work in the machine learning fairness schemes [39]. An additional bias predictor component is attached to the network architecture that helps the encoder module to decorrelate the extracted feature vector from the effects of confounding variables, as evidenced by the classification results for the RBF-Net framework in Section 5. This decorrelation process is based on training the feature encoder through an adversarial learning technique similar to that of the conditional GAN (c-GAN). In this iterative training process, the bias predictor aims to predict the bias value from the feature vector created from a conditioned subset of normal samples from the input and then have an adversarial impact on the encoder to learn the features that are bias- free. In this manner, the RBF-Net framework learned features conditionally independent of the biases and carrying useful information for COVID-19 classification. Thus, the overall goal to create a bias-free and generalizable RD classifier for large-scale clinical deployment can be realized.

The RBF-Net architecture is composed of three key blocks, depicted in Figure 3b, each undergoing distinct training phases. Initially, the COVID-19 classifier block focuses on precise dis ease classification, dynamically updating its model parameters *∂_C_*. The gradient, propagated with respect to the COVID-19 classification loss *L_C_*, refines the encoder parameters *∂_E_*, ensuring feature extraction that minimizes *L_C_*. In the subsequent phase, with frozen encoder parameters *∂_E_*, the bias predictor module is trained. This involves updating parameters *∂_B_* to minimize bias prediction loss *L_B_*, determined by either inverse mean squared error or inverse binary cross-entropy based on the bias variable. In the final training step, with *∂_B_* frozen, the feature encoder undergoes training. Adversarial back-propagation of gradients from *L_B_* fortifies *∂_E_* to extract features maximizing *L_B_*, establishing a min-max game that cultivates bias-invariant features crucial for COVID-19 classification. This strategic training paradigm equips RBF-Net to accurately classify COVID-19 without succumbing to the influence of underlying biased data distributions.

## 4. Methodology

### 4.1. Creation of Cough Spectrograms

In this first stage of the RBF-Net framework, the cough recordings are mapped onto the spectrograms, which depict the spectro-temporal correlations in the audio signal as images. The motivation to use the spectrograms stems from the fact that the spatio-temporal features have the ability to provide comprehensive descriptions of the respiratory sounds (lungs, wheeze, crackles, cough, etc.) [42,43] and to train the deep neural networks [44,45]. The spectrogram is a two dimensional time–frequency representation of the one dimensional cough acoustic samples. The COVID-19 coughs and normal coughs are transformed into the respective time–frequency representations. A spectrogram divides the time domain into several regions. The x-axis characterizes the time domain, while the y-axis represents the frequency domain of the signal. The spectrogram is constructed by a short-time Fourier transform (STFT) with short frame size of 25 ms and stride of 10 ms [46]. Then, a Hamming window of length 2048 is employed that divides the acoustic signal into the frames. To obtain the spectrogram, a 128-point fast Fourier transform (FFT) is implemented on each respective frame. In order to derive the spectrogram final representation, we used the magnitude square of the STFT coefficients. Finally, for achieving computational efficiency, the spectrogram coefficients are log transformed to introduce the compressive nonlinearity and linearly down-sampled onto a 224 × 224 matrix for the cough audio signal.

### 4.2. Biased Training Data Generation

To demonstrate how the biases impact the performance of SoTA DL models and to best evaluate the robustness of the RBF-Net framework, we created multiple training data sets with various biases. In these training data sets, different bias factors were induced pertaining to the following confounding variables: gender, age, and smoking status. In each of these sets of training data, the number of COVID-19 positive and normal class participants were kept equal, to properly address the class imbalance problem. An eminent aspect of this work is that after training models using these sets of biased training data, we also created sets of unseen testing data for their evaluation. The unseen testing data sets were created such that they had a balanced number of participants with respect to each of the confounding variables.

**Figure 4:**
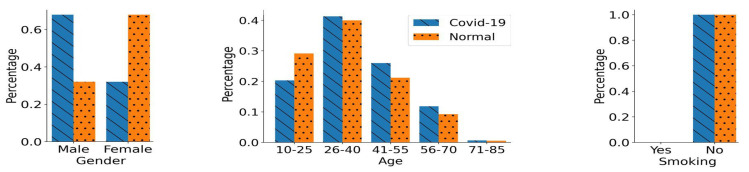
Distribution of the Gender Biased Training Data. In this data set, there is an over-representation of male participants in the COVID-19 class, and there is over-representation of females in the normal class. The age distribution for both classes is identical. Only non-smoking participants were chosen for the creation of this training data set.

(1) Gender Bias:

We created a gender-biased training scenario where the COVID-19 positive rate greatly differs in both male and female gender groups. In this training data set, we have 925 participants in each of the classes, i.e., COVID-19 positive and normal. Although, the total number of participants is balanced in both of the classes, the difference lies in the number of male and female participants in each class (gender bias). As shown in Figure 4, the COVID-19 training class comprises a greater number of male participants compared to the female participants. On the other hand, the normal training class has a greater percentage of female participants. Thus, there exists a bias pertaining to the gender variable in the training data set. Simultaneously, we kept the impact of age and smoking status confounding variables balanced in both of the classes, as shown in Figure 4. We have roughly the same number of COVID-19 and normal participants in each age bracket. Moreover, we only kept the participants with non- smoking status in both classes. Therefore, the bias in the training data set is only induced through gender and not through age and smoking status.

We also created a corresponding gender-based unseen balanced testing data set using the total collected data. In this testing data set, we have 100 participants in each class. We have a balanced number of males and females in both classes, i.e., 50 males and 50 females in both COVID-19 and normal classes. Additionally, we kept the distribution in this testing data set similar to that in the gender-biased training data set in terms of age and smoking status variables.

**Figure 5:**
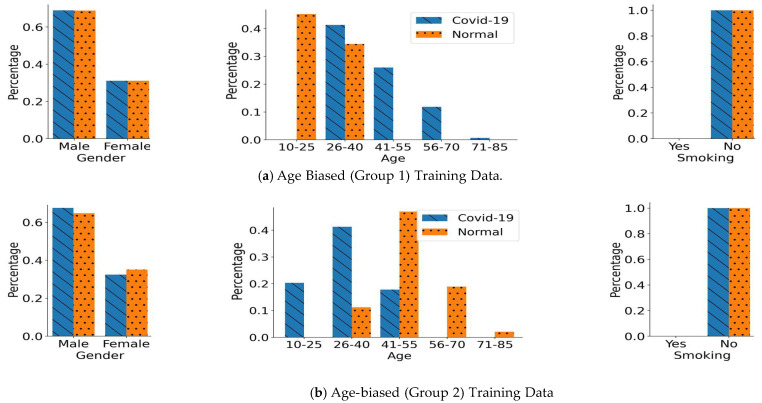
Distribution of the Age-biased Training Data Groups. (**a**) In age-biased group 1, there is an over-representation of relatively younger population (aged under 40) in the normal class compared to the COVID-19 class. (**b**) In age biased group 2, there is over-representation of relatively older population in the normal class (aged above 40) compared to the COVID-19 class. The gender distribution for both classes is identical in these two age-biased groups. Only non-smoking participants were chosen for the creation of both of the training data sets.

(2) Age Bias:

To further study the impact of another unique confounding variable on the performance of DL models, we created age-biased training data sets. For a more generalized evaluation, we created two age-biased training data sets, i.e., age-biased group 1 and age-biased group 2. In both groups, we have 765 participants in each COVID-19 and normal class. The demographics of both age-biased training data sets are shown in Figure 5. In group 1, the normal participants were chosen to be from a relatively younger population i.e., aged under 40 years. At the same time, the COVID-19 class in group 1 included participants from a relatively older population. On the contrary, group 2 has different age distributions in both classes compared to group 1. In group 2, normal class participants were chosen from an older population, i.e., aged above 40 years. Simultaneously, COVID-19 class participants were chosen from a relatively younger population, as shown in Figure 5. In both of these groups, the entire bias is induced only through the age groups. There is no additional bias induced through gender, as the number of male and female participants is approximately equal in each of the classes across both groups. Moreover, only non-smoking participants were chosen in these data sets to nullify the impact of smoking status. Apart from these two training data sets, we also created a corresponding age-based unseen balanced testing data set that includes 100 participants in each of the classes. Both of these classes in the testing data set have an identical age distribution, thus no underlying age bias. Moreover, the testing data set has the same gender and smoking status distribution as the training data sets. Therefore, overall, there is no underlying bias in the unseen balanced testing data set.

**Figure 6:**
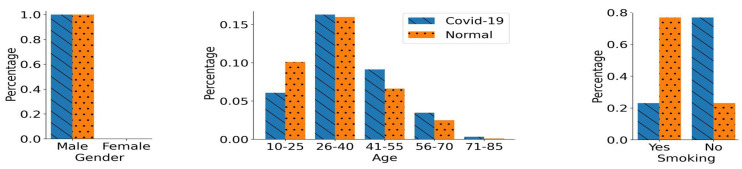
Distribution of Smoking Status-biased Training Data. In this data set, there is an over-representation of non-smoking participants in the COVID-19 class, and there is over-representation of smoking participants in the normal class. The age distribution for both the classes is identical. Only male participants were chosen for the creation of this training data set.

(3) Smoking Status Bias:

For further evaluation of the impact of another distinctive bias on the DL models, smoking status-biased training data sets were also created. Smoking status-biased training data sets contain 350 participants in both the COVID-19 and normal class. However, the total number of participants is balanced in both classes; the difference lies in the number of smoking and non-smoking participants in each class. As shown in Figure 6, the COVID-19 training class comprises a greater number of non-smoking participants compared to the smoking participants. On the other hand, the normal training class has a greater percentage of smoking participants. Thus, there exists a bias pertaining to the smoking status variable in the training data set. Simultaneously, we kept the impact of the other two confounding variables balanced in both classes, as shown in Figure 6. We have approximately the same age distribution in both classes (COVID-19 and normal). Another important aspect of this smoking-biased data set is that as the original cough database comprises more male participants compared to female participants, we only kept the male participants in both classes of smoking-biased training data. Therefore, the bias in the training data set is only induced through smoking status and not through gender and age. Similarly to the other gender and age confounding variables, we created an unseen balanced testing data set for the smoking status as well. In this data set, the number of non-smoking and smoking participants are balanced across both classes. At the same time, this testing data set had the same demographic distribution as the smoking status-biased training data set, which means that there is no innate bias in the smoking status-based unseen balanced testing data set.

### 4.3. Model Training

We trained the CNN-LSTM and the RBF-Net models on the data distributions described in Section 4.2, to analyze the impact of bias in the data distribution on model performance. Both models were implemented in Python 3.6 and Tensorflow 1.21. The training process was accelerated through a high-performance computing cluster integrated with Nvidia V100 Tensor Core GPUs.

To achieve maximum possible accuracy and generalization, we performed extensive hyperparameter tuning on both models. The main purpose was to optimize the values of model parameters that were not learned during training, but rather set before the training process. In the case of CNN-LSTM and the RBF-Net, the hyperparameters that were searched included the learning rate, epochs, batch size, optimizer, and the number of layers/nodes. Hyperparameter optimization was performed by creating small validation data sets having the same data distribution as the biased training data sets. We used these validation sets and the AutoML optimization tool [47] for tuning each model individually. The sensitivity and specificity metrics of the validation sets after the 100th epoch of training were used to identify the optimal hyperparameter configuration. Once these hyperparameters were set, we initiated the training process for both models. The best combination of parameters is listed in Table 1. These CNN-LSTM and RBF-Net models were individually trained using the optimal hyperparameter configuration on each of the biased training sets, followed by their performance being evaluated on the respective unseen testing sets for a fair comparison.

Another crucial aspect of training RBF-Net lies in its convergence and stability. Since RBF-Net fundamentally mimics the c-GAN training scheme for classification of COVID-19 and normal cough sounds, the convergence and stability of the model had to be ensured. The feature encoder of RBF-Net was trained through a min-max game where it aimed to accurately predict COVID-19 while being invariant to the underlying biases. For this reason, the optimal learning rate was very small to avoid fluctuations in the objective loss functions or accuracy metric while training. Moreover, RBF-Net was trained for 1000 epochs, during which the performance metrics during the training and validation process started to stabilize with minimal fluctuations. Once these metrics reached a consistent steady state after approximately 650 epochs, the RBF-Net effectively converged. While training on all training scenarios, the RBF-Net training reached stability and converged for effective COVID-19 prediction without being impacted by confounding variables.

## 5. Results

We trained the CNN-LSTM model on all of the biased training data sets to analyze the impact of confounding effects in the data distribution on this particular SoTA DL model. We utilized 10-fold cross-validation (CV) on the CNN-LSTM model; the data sets were divided into 10 equal folds. In each iteration of CV, one of these folds was used as a validation set, while the other nine folds were used for the training. This process was repeated 10 times, with each fold being used as the validation set once. During each iteration of the CV, we measured the accuracy, specificity, sensitivity, F1-score, and ROC-AUC for the model performance on the validation set. After all iterations were completed, the average of each performance metric across all iterations was calculated and reported. After this CV technique, the CNN-LSTM model architecture was trained on the biased data sets, and its performance was evaluated on the respective unseen balanced testing data sets to gauge if the unrealistic experiment design led to an inaccurate inflation of the model performance. Furthermore, in order to evaluate the efficacy of the RBF-Net framework in learning the features that were purely extracted through difference in cough spectrograms caused by the impact of COVID-19 respiratory disease, we trained it on the biased training data sets as well. We evaluated the performance of RBF-Net on the same unseen balanced testing data sets and performed a comparative analysis with the CNN-LSTM model. In the subsequent subsections, we evaluate the performance of both CNN-LSTM and RBF-Net on each of the biased training data sets individually.

### 5.1. Performance in Gender-Biased Training Scenario

The performance of the CNN-LSTM model in COVID-19 detection was inaccurately inflated under the influence of innate gender bias in the training scenario. From the results obtained through the CNN-LSTM model using the CV technique to the results obtained through the CNN-LSTM on the unseen testing data set, a major decline in performance metrics was observed, as reported in the Table 2. The obtained accuracy dropped from 0.890 to 0.787. This clearly indicated that the CV technique led the CNN-LSTM model to learn the features impacted by the gender bias. Thus, the performance of the CNN- LSTM model in accurately diagnosing COVID-19 was highly overestimated. Another vital observation was the difference in the obtained specificity (0.860) and sensitivity (0.715) of the CNN-LSTM model on the testing data set. Since the samples from the normal class participants were over-represented by the female participants in the training data, the model tended to treat female participants as normal participants. On the other hand, the results of the RBF-Net model, when evaluated on the unseen testing data set, demonstrated a significant improvement over the CNN-LSTM, as measured by the five different performance metrics. We obtained an overall accuracy improvement of more than 5% through the RBF-Net model. The difference between the obtained specificity (0.887) and sensitivity (0.796) was also diminished in the RBF-Net model; thus, it is better suited to mitigate the effect of gender bias in the training scenario.

### 5.2. Performance in the Age-Biased Training Scenarios

Similarly to the gender-biased data, the performance of the CNN- LSTM model in COVID-19 detection was overestimated in the presence of the underlying age distribution in the training scenario. Using 10-fold CV, the CNN-LSTM model obtained an average accuracy of 0.887 and 0.884 in the age-biased groups, respectively, as shown in Table 3. On the other hand, when the CNN-LSTM model was trained on the same training data sets and evaluated on the unseen balanced testing data set, a substantial decline in the performance metrics, such as accuracy, specificity, sensitivity, F1-score, and ROC-AUC, was observed. It achieved an unseen testing accuracy of 0.774 and 0.756 when trained on both groups, respectively. As shown in the previous section (Section 4.2), age-biased group 1 had an over-representation of a relatively younger population (aged under 40 years) in the normal participants. This led the model to treat younger participants as normal samples. This accounted for the significant difference between specificity (0.843) and sensitivity (0.706) obtained through the CNN-LSTM model on the testing data set. Similarly, in age-biased group 2, the normal participants were over-represented by a relatively older population (aged above 40 years). This led the model to treat elderly participants as normal participants, which was again shown by the difference in the obtained specificity (0.825) and sensitivity (0.687). This clearly indicated that CNN-LSTM is prone to learning features directly associated with the underlying age bias, which led to the overestimation of the COVID-19 detection performance. Unlike CNN-LSTM, RBF-Net is immune to the impact of biases in the training data sets. The results for the RBF-Net model demonstrated a major improvement over the CNN-LSTM model in all of the performance metrics when evaluated on the unseen testing data set. An accuracy of 0.845 and 0.818 was achieved across both training groups, respectively, and at the same time, the difference between sensitivity and specificity was also diminished. Thus, it demonstrates that the RBF-Net is also suitable for alleviating the age bias in real-world COVID-19 detection applications.

### 5.3. Performance in the Smoking Status-Biased Training Scenario

The impact of underlying bias in the smoking status distribution on the CNN-LSTM model, causing the inaccurately inflating COVID-19 detection performance, was analyzed. Using a 10-fold CV technique, the CNN-LSTM model obtained an average accuracy of 0.862, as shown in Table 4, whereas, when evaluated on unseen testing data, its accuracy drops to 0.723, which was almost 14% less than that obtained with the CV technique. This clearly shows that the CNN-LSTM model learned features directly associated with the smoking status bias and its COVID-19 detection performance was again overestimated. On the contrary, RBF-Net showed its ability to eliminate the underlying impact of smoking status bias in the training scenario when it was evaluated on the unseen smoking status testing data set. The results for the RBF-Net model showed a major improvement over the CNN-LSTM model in all performance metrics. The obtained accuracy, F1-score, and ROC-AUC were almost 8% higher compared to the CNN-LSTM model.

### 5.4. Ablation Study

To further elucidate the novelty of our proposed approach, which involves integrating the bias predictor module (B) into the CNN-LSTM framework (module A) to address confounding variables, we conducted an additional experiment. In preceding sections, we methodically demonstrated how our RBF-Net (A + B) consistently outperformed the standalone CNN-LSTM model when trained on diverse sets of biased data. In this ablation study, we aimed to assess the specific influence of the bias predictor module (B) within the RBF-Net in comparison to the CNN and CNN-LSTM framework (A) under both unbiased and biased training conditions. The architecture used for constructing the CNN model is illustrated in [27]. To achieve this, we created a new training dataset devoid of any inherent bias related to the confounding variables of age, gender, and smoking status. This dataset consisted of 900 samples for each target class, i.e., COVID-19 and normal. The gender distribution, age distribution, and smoking status distribution were kept roughly the same across both of these classes, thus eliminating any form of bias. Furthermore, we curated an additional balanced unseen testing dataset having 100 samples in both of the classes for evaluating the RBF-Net, CNN, and CNN-LSTM on the unbiased training data. In Table 5 below, we present the testing performance metrics for the CNN model, CNN-LSTM model (Module A) and the RBF-Net (A + B) when trained on the unbiased, gender-biased, age-biased (group 1), and smoking status-biased training datasets and evaluated on their respective unseen testing datasets. This presentation was designed to highlight the discernible impact of the bias predictor (Module B) on the performance of RBF-Net.

The feature encoder module is a shared component in both CNN-LSTM and RBF-Net that plays a fundamental role in learning the distinctive features influenced by the presence of COVID-19. For this reason, the performance achieved by CNN-LSTM and RBF-Net was similar in the unbiased training setting. However, the vitality of the bias predictor (module B) in the RBF-Net was established when both models were trained and evaluated under biased training conditions. In these scenarios, the performance of the CNN-LSTM model experienced a significant decline, whereas the RBF-Net remained resilient, consistently maintaining its accuracy, as highlighted in the preceding sections. Consequently, the incorporation of the bias predictor module (module B) within the c-GAN framework significantly bolstered the RBF-Net’s capability to learn the nuanced impact of COVID-19 features. This enhancement not only contributed to its effectiveness but also rendered it more practical for deployment as a digital testing tool.

## 6. Discussion and Limitations

As discussed in the preceding sections, our investigation delved into the influence of three pivotal confounding variables: gender, age, and smoking status. These variables were meticulously considered to assess their impact on the CNN-LSTM model’s ability to detect COVID-19. Notably, the substantial performance degradation observed in the CNN-LSTM model—from the performance achieved during cross-validation to its subsequent evaluation on the balanced testing data—clearly demonstrated the adverse effects of data selection bias on the features learned by the model. Thus, the underlying demographic bias, that often exists in the distributions of real-world clinical data [34,35,39], has to be addressed in future machine learning schemes, thereby making them practical and effective tools for validation by healthcare practitioners and clinicians in the realm of digital healthcare solutions.

**Figure 7:**
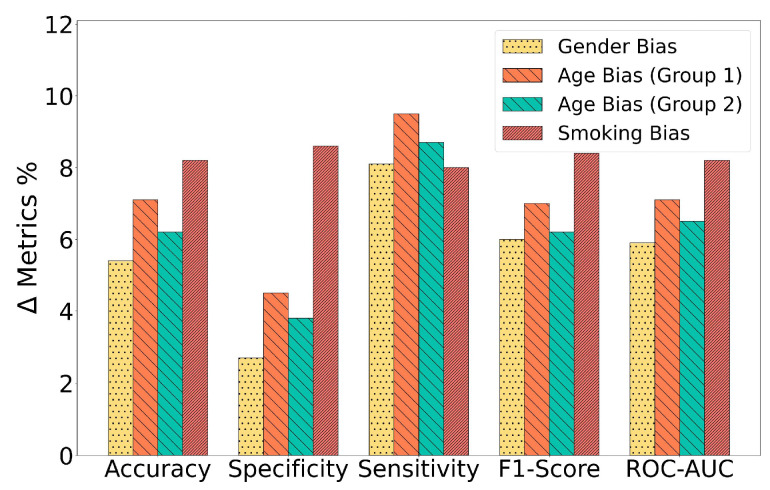
Improvement in the performance metrics (accuracy, specificity, sensitivity, F1-score, and ROC-AUC) achieved by the RBF-Net compared to the CNN-LSTM.

The RBF-Net model effectively mitigated the influence of inherent biases present in training data distributions by extracting meaningful features from them. This was vividly illustrated by the significant performance improvements exhibited by the RBF-Net framework when compared to the CNN-LSTM model across all confounding variables. The differences in performance metrics, including accuracy, specificity, sensitivity, F1-score, and ROC-AUC, across various biased training groups are depicted in Figure 7. A noteworthy insight from the results is that the RBF-Net model achieved the most substantial improvement when dealing with the smoking status-biased training group. It achieved an approximately 8% increase in accuracy, F1-score, and ROC-AUC in this specific scenario. The architecture of the feature encoder module within the RBF-Net sheds light on the reason behind this remarkable improvement. The encoder comprises convolutional blocks and an additional LSTM block, which collectively identify features that hold spatial and temporal significance. This feature extraction capability enabled the RBF-Net model to effectively discern the impact of COVID-19 on cough spectrogram images, as COVID-19 often manifests respiratory symptoms that, in turn, influence the spatiotemporal features of these cough spectrograms, as observed in previous studies [10]. Simultaneously, smoking also induces changes in human cough spectrograms in the spatiotemporal domain. This implies that the effect of smoking on the spectrograms bears some resemblance to the impact of COVID-19, especially when contrasted with the effects of gender and age on the spectrograms. Consequently, the RBF-Net framework yielded the maximum performance increase when mitigating the impact of the bias related to smoking status.

Another standout aspect of our study lies in the high-quality data used for training and testing the DL model, enhancing the credibility of our results and conclusions. Unlike many existing cough-acoustic datasets [27,29,35,48,49], which often rely on crowd-sourced data, the data used in this paper were meticulously collected within a reputable medical facility under the supervision of trained healthcare professionals. Furthermore, the cough audio samples were obtained on the same day as the COVID-19 labels were assigned through standardized RT-PCR tests. This meticulous approach minimized the potential errors stemming from misreporting or participants’ lack of awareness regarding their COVID-19 status at the time of cough recording.

One limitation of our current scope of work is that the efficacy of the RBF-Net framework has thus far been validated solely on the COVID-19 cough dataset and has not yet been extended to other RDs such as tuberculosis, asthma, and COPD. The data collection process for these additional conditions is currently underway within a medical facility, a process that demands a considerable amount of time and resources while adhering to strict Institutional Review Board (IRB) protocols. Additionally, ensuring patient privacy, obtaining ground-truth diagnosis information via gold standard tests at the precise moment of cough sound collection, and maintaining consistency in the cough sound sample collection by minimizing device variability, background noise, and other environmental factors are integral to this endeavor. Our immediate future agenda revolves around evaluating RBF-Net’s capability to identify respiratory diseases akin to COVID-19 based on the acoustic signatures present in cough sounds. This assessment will be conducted once the data collection efforts are successfully concluded.

Our upcoming research will have a bi-fold objective. Firstly, we intend to broaden the spectrum of confounding variables by incorporating the common influenza virus, a respiratory tract ailment. This addition will allow us to explore how the presence of the influenza virus might impact the performance of our RBF-Net in the detection of COVID-19 and other RDs. By doing so, we can unravel potential interactions between these respiratory conditions, thus improving the accuracy of our models. Secondly, we plan to delve into the influence of other common confounding factors, such as race and geographical location. These factors can play a pivotal role in shaping the prevalence of various diseases and introduce biases in data distributions. Examining and accounting for these variables will not only bolster the resilience of the RBF-Net framework but will also make it adaptable for global-scale screening efforts. This comprehensive approach will enhance our understanding of the interplay between various confounding factors and the performance of our model, ultimately facilitating more accurate and globally relevant disease detection and screening.

## 7. Conclusions

This paper addresses the challenge of mitigating underlying bias in training data distributions, which often leads to inflated respiratory disease (RD) diagnosis results. We introduced modifications to the CNN-LSTM architecture to formulate the RBF-Net framework, incorporating an additional bias predictor module. This module assesses the statistical association between the feature vector and biases, aiming to reduce correlations between confounding variables and RD classification outcomes. To illustrate the impact of underlying biases on DL model performance and evaluate the RBF-Net’s effectiveness, we utilized multiple training datasets with various biases (gender, age, smoking status), along with balanced unseen testing datasets. Both the state-of-the-art CNN-LSTM and the proposed RBF-Net models underwent training and evaluation on these datasets. Notably, the RBF-Net framework demonstrated promising and realistic results, even in challenging biased training scenarios, and without relying on cross-validation techniques. These findings suggest the potential of the proposed framework as an effective non-invasive tool for RD testing and screening.

## Figures and Tables

**Table 1:** Optimal Training Parameters for RBF-Net.

Hyperparameters	Best Value
Number of Convolutional Blocks	4
LSTM Units	256
LSTM Activation	tanh
CNN Activation	ReLU
Epochs	1000
Optimizer	ADAM
Batch Size	256
Learning Rate	0.0001

**Table 2:** Performance comparison on the gender-biased training data: Performance evaluation of CNN-LSTM and RBF-Net model in terms of accuracy, specificity, sensitivity, F-1 score, and ROC-AUC.

		Model	Accuracy	Specificity	Sensitivity	F-1 Score	ROC-AUC
	Cross-Validation	CNN-LSTM	0.890	0.913	0.866	0.892	0.893
Gender Bias	Unseen Testing Data	CNN-LSTM	0.787	0.860	0.715	0.785	0.787
		RBF-Net	0.841	0.887	0.796	0.845	0.846

**Table 3:** Performance comparison on both of the age-biased training data sets (Group 1 and Group 2): Performance evaluation of CNN-LSTM and RBF-Net model in terms of accuracy, specificity, sensitivity, F-1 score, and ROC-AUC.

		Model	Accuracy	Specificity	Sensitivity	F-1 Score	ROC-AUC
	Cross-Validation	CNN-LSTM	0.887	0.908	0.867	0.894	0.892
Age-biased Group 1	Unseen Testing Data	CNN-LSTM	0.774	0.843	0.706	0.775	0.776
		RBF-Net	0.845	0.888	0.801	0.845	0.846
	Cross-Validation	CNN-LSTM	0.884	0.901	0.867	0.884	0.881
Age-biased Group 2	Unseen Testing Data	CNN-LSTM	0.756	0.825	0.687	0.757	0.756
		RBF-Net	0.818	0.863	0.774	0.819	0.821

**Table 4:** Performance comparison on the smoking status-biased training data: Performance evaluation of CNN-LSTM and RBF-Net in terms of accuracy, specificity, sensitivity, F-1 score, and ROC-AUC.

		Model	Accuracy	Specificity	Sensitivity	F-1 Score	ROC-AUC
	Cross-Validation	CNN-LSTM	0.862	0.881	0.844	0.867	0.862
Smoking Bias	Unseen Testing Data	CNN-LSTM	0.723	0.750	0.694	0.727	0.723
		RBF-Net	0.805	0.836	0.774	0.811	0.805

**Table 5:** Results of Ablation Studies on RBF-Net.

Model	Unbiased			Gender-Biased			Age-Biased			Smoking Status-Biased		
	Acc	F1-Score	ROC-AUC	Acc	F1-Score	ROC-AUC	Acc	F1-Score	ROC-AUC	Acc	F1-Score	F1-Score
CNN (baseline)	0.821	0.822	0.822	0.751	0.751	0.751	0.746	0.741	0.746	0.683	0.679	0.684
CNN-LSTM (A)	0.874	0.877	0.874	0.787	0.785	0.787	0.774	0.775	0.776	0.723	0.727	0.723
RBF-Net (A + B)	0.879	0.883	0.878	0.841	0.845	0.846	0.845	0.845	0.846	0.805	0.811	0.805

## Data Availability

The statistical data presented in this study are available in Figure 1, Figure 3, Figure 4 and Figure 5. The data sets used and/or analyzed during the current study can be available upon request. These data are not publicly available due to privacy and ethical reasons.
